# The Lighting of Hospitals

**Published:** 1911-02-18

**Authors:** 


### SPECIAL INSTITUTIONAL ARTICLE.

### The Lighting of Hospitals.

### II.—THE LIGHTING OF COTTAGE HOSPITALS AND SMALL SANATORIA.

Where cottage hospitals and sanatoria are within reach of some of the county electric power supply systems it will probably be found convenient to take current from them for the purpose of lighting, and in that case the cost will be found in the same manner as given in the previous article. Gas is usually not to be had in country places, and therefore the establishments referred to have to fall back upon either an electric-lighting plant fixed on the premises, an acetylene gas plant, or one of the new air-gas plants that have been introduced within the last few years. In all cases there will be a certain amount of attendance required; the electric plant will probably require more than either acetylene or air gas, unless there happen to be a man on the establishment who has some knowledge of electricity, and particularly of looking after accumulators. If electric light is decided upon it will be necessary to have an oil engine, a dynamo, and an accumulator, with certain accessories, such as switchboards, in addition to the wires, lamps, and fittings. The accumulator and oil engine also are best fixed in separate buildings away from the hospital buildings proper. The cost of supplying and fixing the oil engine, dynamo, and accumulator, exclusive of the building, may be taken at approximately £2 per light required. The cost of the wires and fittings for the lamps within the hospital may be taken at £1 per lamp; it will vary with the class of fitting and the size of the building.

The cost of acetylene gas plant, with pipes and fittings, will come to about £1 per light. The cost of air-gas plant, pipes, and fittings will range from £1 up to £3 per light, according to the number of lights and the size of the buildings. Both the acetylene gas plant and the air-gas plant should be fixed in special sheds or buildings outside the hospital proper. Acetylene gas is formed by allowing water to drip upon masses of calcium chloride, the water and the calcium chloride being decomposed and the gas given off: this is then usually stored in a gas-holder. The gas-making arrangement is very simple, but, like a great many other things, it requires care in handling, and in particular it is necessary to ensure that there is no chance of an escape of gas into the hospital building. With some forms of acetylene apparatus the manufacture of the gas goes on automatically as it is used up.

Air gas, as it is called, is really a mixture of the vapour of petrol and air. Various machines are employed for the purpose, all of them having the object of securing that the right quantity of petrol vapour is absorbed by the air, that each cubic foot of air receives its proper quantity of petrol vapour, and that the vapour is diffused all through each individual cubic foot. The air gas is burnt just as ordinary illuminating gas, and it owes its illuminating property to the fact that it has not been diluted below a certain figure. It is important that the quantity of vapour present in the air should be kept within certain definite limits, and that the petrol vapour should be confined entirely to the mixing apparatus. Petrol vapour, as is well known, is a very inflammable substance. If allowed to escape into the atmosphere, and to come near a light or a heated body, the vapour ignites, and fires back to the source from which the vapour was obtained.

The running costs of lighting cottage hospitals and sanatoria by electricity will depend upon a number of circumstances. Probably 6d. per Board of Trade unit will not be an excessive estimate to make for the average establishment. This would make the cost of the 25 c.p. metallic filament lamp about the same as the 50 c.p. estimated for in the previous article—22s. 6d. or thereabout for 1,000 hours. Probably the 25 c.p. lamp would give sufficient illumination for cottage hospitals. It might be possible to employ even smaller lamps, but the wisdom of such a course would be doubtful. The cost of acetylene gas is very high. A much smaller quantity of the gas itself is consumed in each burner; but even with this allowance the cost of furnishing the lights works out at a high figure. On the assumption that the same quantity of acetylene gas is consumed in a lamp of a given power as with ordinary illuminating gas in Welsbach mantles, and with acetylene burners giving, say, 20 c.p., and assumed to take 1 cubic foot per hour, the cost works out at about 16s. 6d. for each individual lamp per 1,000 hours. Acetylene gas is burnt in small burners specially constructed for the purpose, and not in the Welsbach mantle. It furnishes a white light, just as the Welsbach mantle does, and of a purer colour; the flame is of the fish-tail shape of the ordinary gas burner, but smaller.

With air gas special forms of Welsbach mantle are employed, and it is claimed that the gas is made for the equivalent of from 2s. to 6s. for 1,000 cubic feet of ordinary coal gas. On the assumption that the mantles take the same quantity of air gas as of ordinary gas, the cost is from 8s. 6d. to 25s. per 1,000 hours. While the low figures mentioned may doubtless be obtained in some cases, the higher figure will rule in others, and the estimate should not be put too low.

